# Latin American Thyroid Society expert panel consensus on anaplastic thyroid cancer

**DOI:** 10.20945/2359-4292-2026-0100

**Published:** 2026-08-31

**Authors:** Inés Califano, Fabiano Mesquita Callegari, Janete Maria Cerutti, Aline Lauda Freitas Chaves, Gilberto de Castro Junior, Jose Miguel Dominguez Ruiz-Tagle, Agustin Falco, Ana Amélia Oliveira Hoff, Luiz Paulo Kowalski, Ana Luiza Maia, Marcos David Pereira, Alvaro Sanabria, Fabian Pitoia

**Affiliations:** 1 Departamento de Endocrinologia, Instituto de Oncologia Ángel H. Roffo, Universidad de Buenos Aires, Buenos Aires, Argentina; 2 Departamento de Patologia, Universidade Federal de São Paulo, SP, Brasil; 3 Departamento de Morfologia e Genética, Escola Paulista de Medicina, Universidade Federal de São Paulo, São Paulo, SP, Brasil; 4 Dom Oncologia e Grupo Oncoclínicas, Divinópolis, MG, Brasil; 5 Instituto do Câncer do Estado de São Paulo, São Paulo, SP, Brasil; 6 Departamento de Endocrinología, Escuela de Medicina, Pontificia Universidad Católica de Chile, Santiago de Chile, Chile; 7 Departamento de Oncología Médica, Instituto Medico Alexander Fleming, Buenos Aires, Argentina; 8 Faculdade de Medicina da Universidade de São Paulo, São Paulo, SP, Brasil; 9 Departamento de Cirurgia de Cabeça e Pescoço e Otorrinolaringologia, A.C. Camargo Cancer Center, São Paulo, SP, Brasil; 10 Faculdade de Medicina, Universidade Federal do Rio Grande do Sul, Porto Alegre, RS, Brasil; 11 Unidad Funcional de Tumores de Cabeza y Cuello, Instituto de Oncologia Ángel H. Roffo, Universidad de Buenos Aires, Buenos Aires, Argentina; 12 Departamento de Cirugía, Facultad de Medicina, Universidad de Antioquia, Centro de Excelencia en Enfermedades de Cabeza y Cuello, Medellín, Colombia; 13 División de Endocrinología, Hospital de Clínicas, Universidad de Buenos Aires, Buenos Aires, Argentina

**Keywords:** Thyroid cancer, anaplastic thyroid cancer, Latin America

## Abstract

Anaplastic thyroid cancer (ATC) is a highly aggressive histologic subtype of thyroid malignancy. Its rarity, rapidly progressive clinical course, along with limitations in health-care resources make management particularly challenging in Latin America. In recent years, substantial advances in the understanding of ATC biology have led to modifications in therapeutic strategies. This consensus aims to raise awareness and provide practical, evidence-based guidance for the diagnosis and management of ATC, summarizing current diagnostic and therapeutic advances while offering an accessible framework tailored to the Latin American context. A multidisciplinary panel of experts in endocrinology, surgery, oncology, pathology, and genetics from Latin American countries developed a series of recommendations. Each recommendation is accompanied by its rationale and supporting evidence, adapted to the regional context to facilitate implementation. All recommendations were reviewed and voted on through a non-anonymous process. Recommendation wording was pre-specified and linked to voting thresholds: “should” indicated unanimous agreement (100%); “is recommended” indicated ≥70% agreement; and “may be considered” was used when agreement was <70% and/or when applicability depended on local availability, feasibility, or patient-specific clinical context. The consensus includes 37 recommendations covering the initial approach, diagnosis, and management of locoregional and advanced disease. By integrating current evidence with expert consensus and accounting for regional resource variability, this document aims to harmonize clinical practice, support multidisciplinary care, and facilitate timely diagnosis and treatment. These recommendations may also serve as a foundation for future research initiatives and the development and updating of regional health policies for this highly lethal disease.

## INTRODUCTION

Anaplastic thyroid cancer (ATC) is a rare and rapidly progressive neoplasia composed of undifferentiated thyroid cells (^1^). Historically, its prognosis was almost uniformly lethal, with a 12-month overall survival (OS) of 21% (^2^). Over recent years, a growing knowledge of its molecular landscape, the wider use of molecular testing, and the introduction of targeted therapies and immunotherapy have significantly transformed the outcomes of this disease for patients receiving optimal care (^3^,^4^). Nevertheless, a large proportion of patients continue to face poor outcomes due to unequal access to a timely diagnosis and advanced treatments. Limited expertise, a scarcity of specialized centers, substantial regional variability in access to molecular testing, and restricted access to state-of-the-art therapies are common challenges in the management of rare diseases. For ATC, effective treatment also requires a swift diagnosis and timely access to appropriate diagnostic and therapeutic interventions. These challenges are further intensified in resource-constrained countries, where patients often face multiple overlapping barriers, including socio-cultural factors and fragile healthcare systems (^5^–^7^).

### Key challenges in the management of ATC in Latin America

- **Geographic barriers:** Most specialized centers are predominantly located in capital and major cities, limiting access for patients in remote or underserved regions.
- **Health care systems disparities:** Marked variability in access to public healthcare, social security and private insurance sectors.
- **Infrastructure gaps:** Diagnostic and therapeutic technologies are not uniformly or readily accessible.
- **Specialists:** Limited availability of dedicated training programs resulting in a reduced number of highly skilled specialists.
- **Limited access to molecular testing:** Constrained by high costs, inconsistent insurance coverage, and uneven regional availability.
- **Limited access to novel therapies:** Driven by high cost, limited reimbursement and, in some cases, lack of regulatory approval.
- **Limited availability of clinical trials:** Most regions have minimal or no participation opportunities.

Addressing such inequities underscores the importance of developing context-sensitive recommendations that target both clinical and systemic gaps. Therefore, this consensus aimed to 1) raise awareness and offer guidance on the current management of ATC, 2) summarize the latest evidence on diagnostic and therapeutic approaches, and 3) provide an accessible framework for diagnosis and treatment that is adapted to the Latin American context and aligned with the need to inform and update health policies.

## MATERIALS AND METHODS

This document presents expert recommendations developed under the auspices of the Latin American Thyroid Society to provide pragmatic, regionally applicable guidelines for the evaluation and management of ATC. Clinical questions were identified by the coordinating group and assigned to 13 panelists according to their areas of expertise. The panel consisted of clinicians and clinician-scientists (five endocrinologists, two surgeons, four oncologists, one pathologist, and one geneticist) who are actively involved in the clinical management and research of advanced thyroid cancers, including ATC. Members represented multiple tertiary referral centers across Latin America, reflecting diverse practice patterns. At the outset, a table outlining six key topics in this field (**Table 1**) was circulated to all panel members, who then prioritized areas of focus. The panel held its inaugural virtual meeting in January 2025.

**Table 1.** Key priority areas addressed in the recommendations

Priority area	Description
Initial evaluation	1.a Emergency evaluation of rapidly growing thyroid masses
	1.b Diagnostic approach and differential diagnosis
	1.c Acquisition of diagnostic material
	1.d Molecular profiling in anaplastic thyroid cancer
Staging and pre-therapy assessment	2.a Multidisciplinary team-based care, therapeutic planning and integration of palliative care
	2.b. Diagnostic imaging, endoscopic procedures, and thyroid neck morbidity scores
Surgical management	3.a The role of surgery for localized disease
	3.b Technical surgical considerations and the role of neoadjuvant therapy
	3.c Elective tracheostomy
External beam radiotherapy (EBRT)	4.a EBRT following R0/R1 resection
	4.b EBRT in unresectable disease
Systemic therapy	5.a “Bridging” therapy
	5.b Dabrafenib plus trametinib for *BRAF*-V600E-mutated anaplastic thyroid cancer
	5.c Neoadjuvant strategies in *BRAF*-V600E-mutated anaplastic thyroid cancer
	5.d Other selective inhibitors (or targeted therapies)
	5.e Cytotoxic chemotherapy and multikinase inhibitors
	5.f Immunotherapy
Other aspects	6.a. Follow-up strategies

Following this meeting, an initial draft was prepared. Each section was authored by one panel member and independently reviewed by two additional members. Recommendations were formulated by interpreting the available evidence within the Latin American healthcare context, including resource availability and regional practice patterns. The draft underwent a full-group review to collect feedback and guide subsequent refinements. All recommendations were discussed and revised through structured virtual meetings and electronic communications. Given that the limited and heterogeneous evidence base for many clinical questions in ATC is predominantly derived from observational studies, small case series, or extrapolation from other settings, we did not apply a formal framework to grade the quality of evidence or the strength of recommendations.

Recommendation phrasing was pre-specified and linked to voting thresholds: “should” indicated unanimous agreement (100%); “is recommended” indicated ≥70% agreement; and “may be considered” was used when agreement was <70% and/or when applicability was expected to depend heavily on local availability, feasibility, or the patient-specific clinical context. All recommendations were voted on in a single round using an open (non-anonymous) voting process. Abstentions were not permitted; therefore, all panelists voted on every recommendation. Recommendations were finalized after incorporating edits aimed at improving wording consistency and operational clarity. The document was then shared with selected Latin American Thyroid Society members for additional input and discussion.

## INITIAL EVALUATION

### Emergency evaluation of rapidly growing thyroid masses

- **Recommendation 1:** A rapidly enlarging thyroid mass should be considered a medical emergency and managed accordingly.
- **Recommendation 2:** In patients with an enlarging neck mass, a rapid and coordinated multidisciplinary approach should be implemented to confirm the diagnosis, evaluate airway safety, and assess disease extent.
- **Recommendation 3:** Sudden changes in differentiated thyroid cancer (DTC) behavior should raise suspicion of transformation to ATC and prompt further evaluation.
- **Recommendation 4:** When ATC coexists with a more differentiated follicular-derived thyroid carcinoma, management should be guided by the anaplastic component.

Rapidly growing thyroid masses, defined as those that enlarge over the course of weeks, should be considered a clinical emergency and managed with a fast-track diagnostic plan. The most likely causes of a rapidly enlarging thyroid mass include ATC and thyroid lymphoma, two aggressive malignancies that require prompt recognition and timely treatment (^8^). For ATC, reducing diagnostic delays has resulted in improved outcomes, allowing patients to participate in clinical trials and improving OS (^9^,^10^). For rapidly growing thyroid masses, the initial approach differs from the routine evaluation of thyroid nodules. Confirmation of the diagnosis, staging, and, in the case of ATC, assessment of the *BRAF* mutational status should be conducted simultaneously and swiftly, ideally within days.

Cross-sectional imaging is essential for accurately assessing the extent of disease. While fine-needle aspiration (FNA) with immediate cytopathologic assessment can support a rapid diagnosis, performing a concurrent core-needle biopsy (CNB) is usually advised to improve diagnostic accuracy and enable molecular profiling (^8^,^11^). Airway compromise is the most urgent concern and must be evaluated at every clinical encounter. Every effort should be made to ensure an expedited diagnosis and management, including hospital admission and early involvement of a multidisciplinary team (^12^,^13^). Prompt referral to experienced centers or consultation with a tumor board should be considered when specialized expertise or technical resources are limited.

A very small subset of DTC undergoes anaplastic transformation, a finding that supports the concept that pre-existing DTC is a risk factor for ATC (reported at around 2% after reoperation for recurrent disease). Risk factors for anaplastic transformation include older age ≥55 years, multiple surgical procedures for recurrent disease (^14^), and, particularly, the co-occurrence of *TERT* promoter mutations with the *BRAF* V600E mutation (^15^). Early recognition of this possibility is critical, despite the risk of overdiagnosis, because timely evaluation and intervention may influence management options and patient outcomes.

The coexistence of both DTC and ATC is a recognized phenomenon. ATC can arise de novo or from DTC and poorly DTC through the progressive accumulation of genomic alterations (^16^). Co-occurring DTC is frequently observed in patients with ATC; this is consistent with a branching evolution during disease development, which may hypothetically occur at any stage of progression (^16^). The clinicopathological characteristics of ATC that develops from DTC remain poorly understood, and the distinct treatment modalities for DTC and ATC further add to this complexity (^17^).

Management of concomitant ATC and DTC remains poorly defined, given the scarcity of evidence and the limited guidance provided in major published guidelines (^13^,^18^–^20^). Because differentiated features characteristic of follicular cells and DTC, including expression of the thyroid-stimulating hormone receptor and the sodium-iodide symporter, are absent in ATC, TSH suppression is not indicated, and radioactive iodine is ineffective and thus not recommended (^13^). There is broad consensus that when an ATC component is present, treatment should primarily target the ATC due to its markedly worse prognosis.

### Diagnostic approach and differential diagnosis of ATC

- **Recommendation 5:** The definitive diagnosis of ATC should be established promptly using FNA cytology and/or CNB, supported by a comprehensive immunohistochemical (IHC) panel to exclude alternative diagnoses.

ATCs are characterized by marked aggressiveness, including frequent extrathyroidal extension, extensive necrosis, high mitotic activity (often atypical), pronounced nuclear pleomorphism, and angiolymphatic invasion (^1^). Morphologically, they are highly heterogeneous and may exhibit epithelioid, spindle cell, giant cell, pleomorphic, paucicellular, rhabdoid, small cell, osteoclast-like giant cell-rich, angiomatoid, or squamous patterns (^1^,^21^). Recognition of these morphological patterns is important because of the broad range of differential diagnoses, although the patterns themselves do not carry prognostic or therapeutic significance. The differential diagnosis includes other primary thyroid malignancies, high-grade lymphomas, melanomas, sarcomas, and, less commonly, metastases (^22^,^23^).

A broad IHC panel is crucial to distinguish ATC from its mimickers (^1^,^22^–^28^) (**Table 2**). In many cases, the most compelling evidence for ATC comes from the lack of expression of markers that would otherwise support other diagnostic possibilities. Cytokeratins (e.g., AE1/AE3, CK7, and CK8/18) are positive in approximately 75% of cases. However, the absence of cytokeratin expression does not exclude ATC (^23^). The thyroid lineage marker PAX8 is positive, at least focally, in 40–60% of cases (^23^). Its expression can support a diagnosis of follicular thyroid cell origin. Thyroglobulin (a thyroid-specific protein) and thyroid transcription factor-1 (TTF-1) are usually negative in ATC, although the latter may show focal or weak positivity in 10–30% of cases (^22^).

**Table 2.** Differential diagnosis of anaplastic thyroid carcinoma

Tumor type	Key distinguishing features	Helpful IHC markers
Poorly differentiated thyroid carcinoma	Intermediate morphology and prognosis between DTC and ATC. The Turin criteria* are useful for diagnosis	Positive: thyroglobulin (focal), TTF-1 (focal) Negative: calcitonin
Medullary thyroid carcinoma	May show focal anaplastic areas mimicking ATC	Positive: Calcitonin, CEA, Chromogranin A, Synaptophysin, INSM-1 Negative: thyroglobulin
High-grade lymphoma	May resemble ATC morphologically. Positive for lymphoid markers, which are absent in ATC	Positive: CD45, CD20 Negative: cytokeratin, thyroglobulin
Intrathyroidal thymic carcinoma (CASTLE)	Composed of squamoid or undifferentiated cells; less pleomorphism than ATC; thymic differentiation	Positive: CD5, CD117 Negative: thyroglobulin
Melanoma	May mimic ATC with pleomorphic epithelioid cells; melanin pigment may be minimal or absent	Positive: S100, SOX10, Melan-A, HMB-45 Negative: thyroglobulin
Sarcomas (primary thyroid or metastatic)	Rare; may resemble ATC morphologically; lack epithelial markers; consider if no pre-existing or co-existing DTC component is identified and/or negative for MAPK mutation (e.g., *BRAF* or *RAS*)	Subtype-specific markers: Angiosarcoma: CD31, ERG-1: Leiomyosarcoma: SMA Rhabdomyosarcoma: Desmin, MyoD1, Myogenin Favor ATC: MAPK mutations (*BRAF, RAS*)
Metastases to the thyroid	Usually in patients with known primary malignancy, morphology may overlap with ATC; lack of thyroid differentiation	Histological correlation with the known primary tumor and use of site-specific immunohistochemical markers are essential to accurately establish the diagnosis Negative: thyroid lineage markers such as thyroglobulin, TTF-1, PAX8 or only focally positive in metastases
Riedel’s thyroiditis	Dense fibrosis replacing normal thyroid parenchyma, often extending beyond the thyroid, chronic inflammatory infiltrate, obliterative phlebitis. Absence of granulomatous inflammation and neoplastic features	Increased number of IgG4-positive plasma cells (≥10 IgG4+ plasma cells per high-power field and an IgG4+/IgG ratio ≥20%) are considered supportive

ATC: anaplastic thyroid cancer; CEA: carcinoembryonic antigen; DTC: differentiated thyroid cancer; IHC: immunohistochemistry; INSM-1: insulinoma-associated protein 1; MAPK: mitogen-activated protein kinase; PAX8: Paired-box gene 8; TTF-1: Thyroid Transcription Factor-1. *Turin criteria include: 1) the existence of a solid/insular/trabecular pattern; 2) the absence of the usual nuclear characteristics of papillary thyroid cancer; and 3) the presence of at least one of the following features: convoluted nuclei, ≥3 mitosis per 2 mm^2^, and tumor necrosis (^29^).

In certain cases, the assessment of proliferative activity based on mitotic figures may be challenging. In these situations, Ki-67 IHC offers a more reliable alternative. ATC typically displays a high Ki-67 proliferation index, often ≥30% (^23^). In addition, p53 often shows an aberrant IHC pattern in approximately 50–60% of ATCs, suggesting a somatic *TP53* mutation; this presents as either a complete loss of p53 protein expression or markedly increased p53 protein abundance due to an extended protein half-life (^1^,^20^).

True squamous differentiation, along with immunoexpression of p63, p40, and CK5/6, can be seen in ATCs. In the current World Health Organization Classification (5th Edition), squamous cell carcinoma of the thyroid is considered a subtype of ATC (^1^,^20^). Because the pooled incidence of the *BRAF* V600E mutation is 33% (range, 0–93%) in ATCs, IHC for the *BRAF* V600E mutation using the VE1 antibody may serve as a surrogate for molecular testing in the preoperative setting or immediate post-diagnostic period, particularly for guiding targeted BRAF inhibitor therapy (^20^). However, IHC is an imperfect predictor of mutation status; therefore, positive results should be confirmed by molecular testing whenever possible (^23^).

### Acquisition of diagnostic material

The first-line method for obtaining material from a neck mass is ultrasound-guided FNA (^23^). FNA performs well as a screening test, demonstrating a sensitivity of 100% and a positive predictive value of 89% (^29^). This is a rapid, safe, and cost-effective procedure capable of providing a preliminary morphological diagnosis within minutes (^30^). Multiple needle passes should be performed in different areas of the lesion to ensure the sampling of viable tumor cells and to avoid sampling only necrotic areas. Recent advances in molecular techniques allow for the reliable detection of common driver mutations, such as *BRAF* V600E*, RAS*, and *TERT* promoter mutations, even in small cytologic samples (^31^).

For each pass, part of the material is used to prepare smears, while the remainder can be allocated to cell block preparation. To optimize the quality of the cell block for IHC, some dedicated passes should be performed specifically for this purpose. Whenever feasible, a CNB should be performed concurrently with the FNA. This approach provides complementary tissue for histological diagnosis and ancillary studies, including comprehensive genomic testing (e.g., DNA- or RNA-based next-generation sequencing [NGS] panels) (^11^).

As with FNA, CNB samples may be nondiagnostic if only fibrotic or necrotic tissue is obtained (^32^); ultrasound (US) plays a critical role in identifying solid, viable areas for targeted sampling (^11^). When available, rapid on-site evaluation by either a cytotechnologist or a pathologist is highly beneficial for confirming sample adequacy during the procedure (^33^). In emergency settings, especially for patients presenting with respiratory compromise, an open biopsy may be performed during a tracheostomy.

### Molecular profiling in ATC

- **Recommendation 6:** Rapid *BRAF* V600E testing should be performed as soon as possible after diagnosis (ideally within 24 hours) when targeted therapies are available and timely testing is feasible. While molecular testing is preferred when accessible, IHC may be used as an initial rapid screening method, followed by molecular confirmation whenever feasible.
- **Recommendation 7:** More comprehensive molecular profiling should be performed whenever targeted therapies or clinical trials are available. In settings where available, targeted NGS panels are recommended for both targeted and broad detection of actionable alterations.
- **Recommendation 8:** Consider obtaining blood for a liquid biopsy when adequate tumor tissue is unavailable, as it represents a less sensitive but minimally invasive alternative for molecular testing.

Because ATC often exhibits marked genomic heterogeneity, broad NGS panels are preferred, as they enable the detection of coexisting driver mutations, rare fusions, and biomarkers relevant to targeted therapy or immunotherapy decisions, such as tumor mutational burden and microsatellite instability. Alternatively, when NGS is not feasible, selected actionable alterations may be assessed using targeted assays, provided that corresponding therapies and/or clinical trials are available. The main genetic alterations in ATC are summarized in **Table 3**.

**Table 3.** Genetic alterations in ATC: prevalence and testing methods

Genetic alteration	Prevalence	Testing method	Notes
*BRAF* V600E	0–93%	IHC, Sanger Sequencing, PCR*, DNA-based NGS panel	Most common actionable driver: Urgent reflex testing for *BRAF* V600E is critical Associated with rapid and durable treatment responses
*RAS* (*HRAS/KRAS/NRAS*)	9–50%	Sanger Sequencing, PCR, DNA-based NGS panel	No approved direct inhibitors; prognosis and trial-eligibility implications
*TERT* promoter (C228T/C250T)	30–75%	PCR*, Sanger sequencing, DNA-based NGS panel	Strong predictor of poor outcomes; genetic duet with *BRAF* or *RAS* further worsens prognosis
*ROS1* fusions	Rare	PCR*, Sanger sequencing, RNA-based NGS panel	Case reports show responses to selective inhibitors
*ALK* fusions	<5%	FISH, RT-PCR, RNA-based NGS panel	Case reports show responses to selective inhibitors
*NTRK* fusions	<5%	FISH, RT-PCR, RNA-based NGS panel	FDA-approved therapies for any tumor with *NTRK* fusion
*RET* fusions	<5%	FISH, RT-PCR, RNA-based NGS panel	FDA-approved therapies for *RET*-driven thyroid tumors
*PTEN* mutation	0–18%	DNA-based NGS panel	Off-label therapies, limited efficacy
*TP53*	40–80%	IHC, Sanger sequencing, NGS panel	Not targetable
*PIK3CA* mutation	0–25%	Sanger sequencing, DNA-based NGS panel	No approved targeted therapies; potential eligibility for clinical trial
High tumor mutation burden	10–20%	Broad NGS	Predicts response to immunotherapy; important when driver mutations are absent; tumor-agnostic approval
Microsatellite stability-high (MSI-H)	<5%	Broad NGS	Predicts response to immunotherapy; important when driver mutations are absent; tumor-agnostic approval

IHC: immunohistochemistry; *Single gene PCR (allele-specific, digital PCR); NGS: next generation sequencing.

When primary tumor tissue is unavailable or inadequate, and the sampling of recurrent or metastatic disease is unfeasible or insufficient, liquid biopsy using circulating tumor DNA and/or circulating tumor RNA may serve as a minimally invasive alternative for the rapid detection of actionable point mutations or fusions, respectively (^34^). While less sensitive than tissue-based assays, this approach may better capture tumor heterogeneity and the broader spectrum of genomic alterations.

ATC is characterized by a complex genomic landscape with a broad spectrum of somatic mutations and a generally moderate mutational burden. These alterations can be grouped into early mutations, driver events activating the mitogen-activated protein kinase (MAPK) and PI3K/AKT pathways, which are also commonly found in DTC, and late mutations, such as *TERT* promoter and *TP53* mutations, which play a critical role in anaplastic transformation (^20^,^35^–^37^).

Although the* BRAF* V600E mutation is one of the most frequent early alterations identified in ATC (^20^,^38^), mutations in *RAS* genes (*HRAS, KRAS*, and* NRAS*) are also frequently observed (^20^,^38^). Multiple studies have shown that *RAS* mutations are associated with poorer survival outcomes (^36^). While the* BRAF* V600E mutation represents the most prevalent and clinically actionable MAPK driver, other MAPK-related fusions, including *RET, ALK,* and *NTRK*, are also clinically actionable when detected (^20^,^23^,^35^,^39^–^41^).

Alterations in tumor suppressor genes and components of the PI3K/AKT/mTOR signaling pathway are more frequently observed in ATC than in PTC, contributing significantly to its aggressive phenotype. Overall, genetic alterations in the PI3K/AKT/mTOR pathway can be found in 40–57% of ATC patients (^20^,^35^,^38^). Mutations in *PIK3CA* are found in up to 25% of ATC cases, while *PTEN* mutations or loss of expression are reported in up to 18% of cases. These alterations represent potential therapeutic targets; consequently, agents inhibiting various components of this pathway, such as mTOR inhibitors, have been explored in several small clinical trials (^39^).

These mutations also hold significant prognostic importance, as they are linked to adverse outcomes such as metastatic dissemination and poorer OS in ATC patients. Furthermore, *BRAF* V600E mutations frequently cooperate with alterations in the PI3K/AKT/mTOR pathway, including *PIK3CA* mutations. This co-occurrence drives aggressive tumor phenotypes, decreases survival, and contributes to resistance to BRAF inhibitors (^20^).

Loss-of-function mutations in the *TP53* gene are exceptionally common in ATC, making them one of the most prevalent genetic alterations. With reported frequencies ranging from 42.6% to 75%, these mutations are strongly associated with genomic instability and a poor prognosis (^35^). Copy number loss in the tumor suppressor genes *CDKN2A*/2B suggests that these alterations may the underlying drivers of disease progression and dedifferentiation (^37^).

*TERT* promoter mutations (C228T and C250T) are highly prevalent in aggressive thyroid cancers, particularly in ATC, where frequencies can reach up to 75% (^20^,^38^,^40^). These mutations are strong independent prognostic markers for poor outcomes, including distant metastasis and increased disease-specific mortality. The coexistence of *TERT* promoter mutations with *BRAF* or *RAS* mutations is associated with a worse prognosis, highlighting a “genetic duet” that confers heightened aggressiveness (^20^,^40^).

## STAGING AND PRE-THERAPY ASSESSMENT

### Multidisciplinary team-based care, therapeutic planning and integration of palliative care

- **Recommendation 9:** ATC management should be delivered by a multidisciplinary team, with attention to both curative-intent strategies and palliative goals, alongside the active engagement of patients and their families or caregivers to ensure holistic care.
- **Recommendation 10:** Due to the poor prognosis and limited evidence guiding many management decisions in ATC, special consideration should be given to patient preferences after thorough discussion.

A coordinated multidisciplinary approach is essential in ATC, given the disease’s aggressiveness, historically poor prognosis, and recent therapeutic advances that can meaningfully extend survival. Multidisciplinary tumor board evaluation within days of diagnosis is vital to ensure timely and coordinated care. Teams should rapidly clarify the goals of care, whether therapeutic or palliative (^4^,^13^). Treatment planning constitutes a medical emergency that necessitates individualized approaches. One model is MD Anderson’s Facilitating Anaplastic Thyroid Cancer Specialized Treatment program, a rapid-access pathway designed to accelerate diagnosis, molecular profiling, and treatment through the integration of a group of endocrinologists, oncologists, head and neck surgeons, pathologists, radiologists, radiation oncologists, and palliative care specialists (^3^,^4^). Most patients require combined treatment modalities tailored to their disease stage and performance status, while supportive care and airway evaluation must remain priorities. Continuous multidisciplinary oversight is necessary to monitor treatment response, manage toxicity, and adapt strategies as the disease evolves.

Patients and their families should receive clear and timely prognostic information, along with support to weigh the risks and benefits of available options (^12^,^13^). Realistic expectations should be established, and patient preferences regarding the extent of aggressive interventions must be discussed and respected. In the context of advanced disease, certain therapies (e.g., chemotherapy) may reduce quality of life and increase healthcare costs without improving survival.

Early discussions are therefore essential, covering topics such as airway management (and the potential need for a tracheostomy), nutritional support, hospice care, and end-of-life preferences, including symptom control for pain and dyspnea (^13^,^42^). Palliative care should be integrated from the time of diagnosis to address symptoms, psychosocial needs, and family support. Depending on the disease trajectory and patient values, care may combine active therapy with supportive measures or transition to exclusively palliative approaches. Failure to discuss end-of-life considerations may lead to a poor prognostic understanding, consequently impairing shared decision-making and delaying referral to palliative care services (^43^).

### Diagnostic imaging, endoscopic procedures, and thyroid neck morbidity scores

- **Recommendation 11:** Cross-sectional imaging of the neck (contrast-enhanced computed tomography [CT] or magnetic resonance imaging [MRI]) should be performed in all patients diagnosed with ATC to comprehensively assess the extent of the tumor and its invasion into adjacent structures.
- **Recommendation 12:** To thoroughly assess disease extension, an ^18^F-FDG PET/CT is recommended for all patients diagnosed with ATC. If it is not readily available, a contrast-enhanced CT of the neck, thorax, abdomen, and pelvis should be conducted to avoid delaying treatment.
- **Recommendation 13:** A contrast-enhanced brain MRI is recommended at the diagnosis of ATC to ascertain the presence of brain metastases. Additionally, it should be performed in the presence of suggestive clinical findings.
- **Recommendation 14:** An assessment of vocal cord mobility, ideally via laryngoscopy, should be performed in all patients diagnosed with ATC to assess vocal cord function and potential tumor involvement of the larynx and upper trachea.
- **Recommendation 15:** Upper gastrointestinal endoscopy is recommended at the diagnosis of ATC in patients with clinical or radiographic evidence suggesting upper gastrointestinal tract involvement.
- **Recommendation 16:** Bronchoscopy is recommended at the diagnosis of ATC in patients with clinical or radiographic evidence suggesting tracheal involvement.

Given the aggressive nature of ATC, comprehensive imaging is essential for accurately defining disease extent and guiding management. Several imaging modalities may be used depending on availability. Local invasion is almost universal and must be carefully characterized for treatment planning (^44^,^45^). Distant metastases are present in up to 50% of patients, most commonly involving the lungs, bones, and brain (^44^–^48^).

Cross-sectional imaging of the neck, using CT or MRI, assesses the local extent of the tumor and its invasion into adjacent structures, which is essential for surgical planning and evaluating resectability (^4^,^13^,^49^–^51^). An ^18^F-FDG PET/CT scan provides a detailed assessment of both local and distant disease involvement. It is particularly effective in detecting metastases that might be missed by other imaging techniques, significantly influencing management strategies by identifying previously unknown metastatic sites (^4^,^44^,^52^,^53^). Although an ^18^F-FDG PET/CT is considered the test of choice for staging, its availability in most Latin American centers is limited or nonexistent. Even in centers where it is available, its use is frequently delayed due to health system–related barriers. In settings where an ^18^F-FDG PET/CT is inaccessible, a contrast-enhanced CT or MRI of the neck, chest, abdomen, and pelvis represents a practical alternative, given its broader availability (^4^,^13^). Additionally, if bone metastases are detected, further evaluation (e.g., a spine MRI in the case of vertebral metastases) is advised to characterize the lesions more thoroughly (^4^,^13^).

With brain metastases present in up to 5% of cases, the utility of universal brain imaging remains debated. Nevertheless, given its potential to influence management, brain imaging is advised at diagnosis for all patients and is mandatory in the presence of symptoms suggestive of neurological involvement (e.g., headaches or neurological deficits). MRI is preferred over CT due to its greater sensitivity for detecting brain metastases (^4^,^12^,^13^).

In ATC, the risk of laryngeal nerve involvement and subsequent vocal cord paralysis necessitates meticulous surgical planning, underpinned by a thorough assessment of vocal cord function (^4^,^13^). The preferred evaluation method is a laryngeal assessment via fiberoptic laryngoscopy, which provides a comprehensive examination of vocal cord mobility and can detect direct tumor involvement of the larynx and upper trachea (^4^,^13^,^54^). In scenarios where fiberoptic laryngoscopy is unavailable, direct and indirect laryngoscopy or a transcutaneous laryngeal US may serve as alternative diagnostic tools. While a transcutaneous laryngeal US is typically employed in the postoperative evaluation of thyroidectomy patients, it represents a viable option for assessing vocal cord function in this context. Nevertheless, a limitation of this modality is that most patients present with a high tumor volume, which obscures the vocal cords and prevents the use of alternative viewing windows, such as a lateral approach (^13^,^55^,^56^).

Upper gastrointestinal endoscopy and bronchoscopy are not typically included in the initial staging protocol for ATC. Nevertheless, they become pertinent investigations in instances of clinical or radiographic evidence suggesting airway or upper gastrointestinal tract involvement or bleeding. In such cases, endoscopic findings can significantly influence the management strategy, guiding therapeutic decisions appropriate to the patient’s condition (^4^,^13^,^57^).

## SURGICAL MANAGEMENT OF ATC

- **Recommendation 17:** Surgery should be offered exclusively when R0 or R1 resection is feasible, and it should be followed by comprehensive adjuvant multimodal therapy.
- **Recommendation 18:** For stage IVA and IVB disease, upfront surgical intervention should remain standard practice when anatomically and functionally appropriate.
- **Recommendation 19:** R2 (debulking) surgery should be strictly avoided due to a lack of clinical benefit and increased patient morbidity.
- **Recommendation 20:** Neoadjuvant targeted therapy should be strongly considered for patients with *BRAF* V600E mutations presenting with initially unresectable disease.
- **Recommendation 21:** For patients with *BRAF* V600E-negative ATC and initially unresectable diseases, alternative neoadjuvant therapies should be considered when actionable alterations are identified by molecular profiling.
- **Recommendation 22:** In patients with stage IVC ATC, surgery is generally not recommended; however, it may be considered in exceptional cases presenting with a limited systemic disease burden, or for symptom management.
- **Recommendation 23:** Elective tracheostomy should not be routinely performed.

### The role of surgery for localized disease

Most patients are not candidates for upfront surgery due to local disease extension. For individuals presenting with resectable stage IVA or IVB disease, surgical intervention represents a cornerstone of potentially curative treatment. The recommended approach involves a total thyroidectomy accompanied by central compartment lymph node dissection, with selective lateral neck dissection based on the extent of nodal involvement. However, surgery alone is insufficient, and all patients require adjuvant postoperative therapy, including intensity-modulated radiation therapy and systemic treatment (^13^,^58^).

### Technical surgical considerations and the role of neoadjuvant therapy

When evaluating a patient with ATC, operability should be assessed first, followed by resectability. Operable patients are those without extensive metastatic disease and are fit for major surgery. Surgery should be performed only when complete macroscopic tumor removal is feasible and an R0 or, at most, R1 resection is realistically achievable. Debulking (R2) resections provide no survival benefit, significantly increase morbidity, and may delay the initiation of systemic therapy (^13^,^58^–^61^).

Tumors invading the prevertebral fascia, brachial plexus, neck or mediastinal vessels, or requiring non-reconstructable tracheal or esophageal resection are generally considered unresectable (^62^). Surgical management of ATC requires highly specialized expertise and multidisciplinary planning frequent invasion of critical cervical structures. Extension to the trachea, esophagus, or major vessels may demand complex *en bloc* resections performed in collaboration with thoracic, vascular, or reconstructive surgery teams (^13^,^63^). However, radical procedures are usually discouraged and reserved for selected cases. Many patients are better candidates for molecularly guided neoadjuvant therapy, with regular reassessment to determine potential conversion to resectability or, failing this, transition to palliative care (^64^,^65^). Ongoing controversy about upfront surgery versus neoadjuvant approaches has prompted attempts to standardize operability criteria, utilizing tools such as the Thyroid Neck Morbidity Complexity and Surgical Morbidity Complexity Scores (^66^,^67^).

The identification of actionable genetic alterations, particularly the* BRAF* V600E mutation, has led to a paradigm shift in the management of unresectable ATC. In tumors harboring targetable mutations, neoadjuvant targeted therapy may downstage previously inoperable disease, enabling surgical resection and potentially improving both local control and survival. This strategy has produced what is likely the most important and transformative change in clinical practice to date (see the next sections on BRAF inhibitors used alone or in combination with immunotherapy) (^66^,^68^). During neoadjuvant treatment, the patient should be regularly re-evaluated using contemporary imaging to assess tumor response and the possibility of conversion to a less aggressive surgical procedure. When tumor resectability remains uncertain, the surgeon may perform intraoperative staging to assess invasion of major vessels or the upper aerodigestive tract.

Patients with stage IVC disease and distant metastases present unique challenges. In this setting, surgical intervention is generally reserved for palliative purposes, aimed at addressing life-threatening complications (e.g., airway obstruction, uncontrolled bleeding, or severe dysphagia). While curative resection remains uncommon in metastatic disease, carefully selected patients with limited metastatic burden and technically feasible primary tumor resection may experience modest survival benefits when surgery is incorporated into a comprehensive multimodal treatment plan (^59^–^61^,^69^).

### Elective tracheostomy

Given that nearly all patients with ATC are at risk for airway obstruction, elective tracheostomy was historically considered a palliative option. Nevertheless, tracheostomy significantly decreases quality of life, and its impact on survival prolongation is limited. Bleeding and tumor growth through the incision are frequent complications. Consequently, a paradigm shift has occurred over the past decade, and currently, prophylactic tracheostomy is not recommended (^42^). However, the potential need for a procedure in the event of acute airway distress must be discussed. Emergency tracheostomy is technically demanding and should be performed in an operating room by experienced head and neck surgeons under general anesthesia. Emergency tracheostomy conducted for symptomatic airway obstruction carries a 30-day mortality rate of 17.6% (^70^).

## EXTERNAL BEAM RADIOTHERAPY (EBRT)

- **Recommendation 24:** Postoperative EBRT should be indicated for all patients with ATC after R0/R1 resections.
- **Recommendation 25:** Radiosensitizing chemotherapy is recommended for all patients receiving EBRT after R0/R1 resections.
- **Recommendation 26:** EBRT should be a part of the therapeutic approach of unresectable ATC as it enhances regional control and palliates symptoms.

### EBRT following R0/R1 resections

Local complications are a leading cause of morbidity and mortality in ATC (^71^,^72^). Improving locoregional control remains a critical component of ATC management, even after satisfactory surgical resection. The role of adjuvant therapy (radiation or chemoradiation) has been evaluated predominantly in retrospective series, which included heterogeneous populations, varying radiation doses, and combinations with different radiosensitizers.

Despite these limitations, multiple studies and a 2016 meta-analysis demonstrate that adding postoperative radiotherapy or chemoradiation to surgery for stage IVA/IVB ATC improves local control and approximately doubles OS compared to surgery alone, especially in patients who underwent R1/R2 resection. These findings were confirmed in National Cancer Database analyses, where multimodal treatment (surgery plus chemoradiation) increased 1-year OS from approximately 13–22%, with the greatest benefit observed in patients who had R0/R1 resections (^73^–^75^).

Very low-dose doxorubicin is the most studied radiosensitizer in ATC, with combinations with EBRT showing improved response rates and survival (^76^,^77^) but raising concerns regarding toxicity. Weekly taxanes (docetaxel, paclitaxel) are currently the most frequently used radiosensitizers (^78^). However, OS remains poor. The addition of immunotherapy or tyrosine kinase inhibitors to chemoradiation has shown no clear benefit and is not recommended outside clinical trials (^79^,^80^).

### EBRT for unresectable disease

For patients with locoregionally confined but unresectable ATC, EBRT, alone or in combination with systemic therapy, remains a cornerstone of treatment, particularly when neoadjuvant systemic strategies are not feasible. EBRT can provide durable locoregional control and palliate symptoms such as pain, dysphagia, or airway compromise (^13^,^58^).

Given the rapid progression of ATC, hypofractionated EBRT regimens, which deliver higher doses per fraction over a shorter overall treatment duration, have been investigated. Hypofractionated EBRT has demonstrated non-inferior OS compared to conventional fractionation, with manageable toxicity. Dose escalation to ≥50 Gy has been associated with improved OS, and higher radiation doses (≥60 Gy) have been correlated with better outcomes, even in the absence of surgery (^81^–^83^). These findings support the integration of hypofractionated EBRT into multimodal treatment approaches for select patients, particularly those with good performance status and without significant metastatic burden (^84^–^86^).

Concurrent chemoradiotherapy can further enhance locoregional control and may, in rare cases, enable surgical resection of initially unresectable tumors. Patients who have undergone R2 resection or present with unresectable but non-metastatic disease and are suitable candidates for aggressive therapy should be considered for intensity-modulated radiation therapy with concurrent systemic therapy (^13^,^87^).

For patients with advanced, symptomatic, or metastatic disease not amenable to curative treatment, palliative EBRT is a key component of supportive care. Administered at lower doses over shorter periods, palliative EBRT can effectively alleviate airway obstruction, bleeding, or pain, thereby improving quality of life. Treatment decisions should be individualized, taking into account performance status, care objectives, and anticipated toxicity.

Overall, EBRT, whether with curative or palliative intent, plays a critical role in the multidisciplinary management of ATC, with hypofractionated approaches offering a promising balance between efficacy, treatment duration, and tolerability.

The technical aspects of radiotherapy for ATC are summarized below.

### Principles of external beam radiotherapy in ATC

#### Dose

- Doses <50 Gy are not recommended.
- Doses >60 Gy are associated with improved OS.

#### Fractionation

- 60 Gy in 2 Gy fractions is the most standard regimen.
- Hyperfractionated regimens are not recommended.
- Hypofractionated regimens may be considered in patients with poor performance status.

#### Target volume

- The tumor bed and cervical lymph nodes, including cervical and upper mediastinal areas, should be included.
- Level I, retropharyngeal, and retrostyloid regions are not routinely included.

#### Technique

- Intensity-modulated radiation therapy is the preferred technique.
- Lack of advanced planning techniques should not delay treatment.

#### Radiosensitizing chemotherapy

- Taxanes are the most frequently used agents.
- Other agents may also be considered.

## SYSTEMIC THERAPIES IN UNRESECTABLE NON-METASTATIC/METASTATIC DISEASE

### “Bridging” therapies

- **Recommendation 27:** In patients who wish to pursue an intensive therapeutic approach, bridging therapies (cytotoxic chemotherapy or lenvatinib) are recommended when delays in initiating more effective targeted treatments are anticipated.

Due to the imminent threat of ATC progression, starting treatment without delay is a critical discussion point for patients desiring a more intensive approach. While awaiting molecular results or access to targeted therapies, the use of cytotoxic agents (such as taxanes, platinum compounds, and/or doxorubicin) (**Table 4**) as an initial “bridging” therapy represents a reasonable approach.

**Table 4.** Systemic pharmacological agents commonly employed in the management of anaplastic thyroid cancer (ATC)

Selective inhibitor	Description
*BRAF* V600E mutated ATC	Dabrafenib 150 mg PO twice daily and trametinib 2 mg PO daily
*NTRK* fusion-positive ATC	• Larotrectinib 100 mg PO twice daily • Entrectinib 600 mg PO daily
*RET* fusion-positive ATC	Selpercatinib 160 mg PO twice daily
*ALK* fusion-positive ATC	Crizotinib 250 mg PO twice daily
Systemic chemotherapy (combined regimes)	• Paclitaxel/carboplatin: • Paclitaxel 60–100 mg/m^2^ IV, carboplatin AUC 2 IV (weekly) Paclitaxel 135–175 mg/m^2^ IV, carboplatin AUC 5–6 IV (every 3–4 weeks) • Docetaxel/doxorubicin: • Docetaxel 20 mg/m^2^ IV, doxorubicin 20 mg/m^2^ IV (weekly) • Docetaxel 60 mg/m^2^ IV, doxorubicin 60 mg/m^2^ IV (with G-CSF) (every 3–4 weeks) (4) • Doxorubicin/cisplatin: • Doxorubicin 60 mg/m^2^ IV, cisplatin 40 mg/m^2^ IV (every 3 weeks)
Systemic chemotherapy (monotherapy)	Paclitaxel 60–90 mg/m^2^ IV (weekly) or 135–200 mg/m^2^ (every 3–4 weeks) Doxorubicin 20 mg/m^2^ IV (weekly) or 60–75 mg/m^2^ IV (every 3 weeks)
Radiosensitizing chemotherapy	• Paclitaxel 30–60 mg/m^2^ IV weekly • Docetaxel 20 mg/m^2^ IV weekly
Immunotherapy	Pembrolizumab 200 mg IV every 3 weeks
Other possible agents	
Lenvatinib/pembrolizumab	Lenvatinib 20–24 mg PO daily and pembrolizumab 200 mg IV every 3 weeks
Pemetrexed/carboplatin	Carboplatin AUC 5-6 plus pemetrexed 500 mg/m^2^ IV every 3 weeks

AUC: area under the curve; G-CSF: granulocyte colony-stimulating factor; IV: intravenous; PO: per os (oral administration).

Objective response rates can be achieved in up to 21% of cases, with disease control rates of up to 64% and a median OS of approximately 6 months. Although these outcomes are modest, they may stabilize the disease and prevent further progression while awaiting the initiation of more effective targeted therapies (^78^). Lenvatinib may also be used as an alternative bridging therapy (^88^). Partial responses, or stable disease with minor responses to a rapid improvement in respiratory symptoms, have been reported in a small number of patients. The risk of fistula formation in patients with ATC should be carefully considered (^89^).

### Dabrafenib/trametinib for *BRAF*-mutated ATC

- **Recommendation 28:** Dabrafenib/trametinib (DT) should be promptly initiated as the first-line systemic therapy for unresectable or metastatic *BRAF* V600E-mutated ATC, either as a single strategy or in combination with immunotherapy.
- **Recommendation 29:** Restaging with contrast-enhanced CT is recommended after 1–3 months of DT therapy to reassess the feasibility of surgical resection.
- **Recommendation 30:** When DT is unavailable, other BRAF inhibitors may be considered as an alternative.

The *BRAF* V600E mutation represents the most common actionable genetic alteration in ATC (^90^). Clinical evidence supports the efficacy of dabrafenib (a BRAF inhibitor) and trametinib (a MEK inhibitor), collectively termed DT, in treating *BRAF* V600E*-*mutated ATC (**Table 3**). The 2017 Rare Oncology Agnostic Research (ROAR) basket trial demonstrated an initial objective response rate (ORR) of 69% in 16 patients, including one complete response, and a one-year OS rate of 80% (^91^). The US Food and Drug Administration (FDA) approved DT in 2018 for locally advanced or metastatic ATC with a *BRAF* V600E mutation when there are no satisfactory locoregional options (^92^). A six-year follow-up revised the ORR to 56%, with three complete responses, a median progression-free survival (PFS) of 6.7 months, and a median OS of 14.5 months (^93^).

Real-world data on ATC are scarce. Small UK and Argentinian series suggest modest survival and variable responses to DT. A large Spanish/Portuguese registry (214 pts) reported a median OS of 4.5 months, with access to NGS and *BRAF* mutation status as key prognostic factors (^94^–^96^).

### Neoadjuvant approach to *BRAF*-mutated ATC

R0/R1 resection is a key prognostic determinant of OS in ATC and should remain the therapeutic goal, either at initial presentation or after neoadjuvant treatment. Responses to DT are typically rapid and robust, yet short-lived, prompting the use of a neoadjuvant approach. In unresectable *BRAF-V600E-mutated ATC,* DT has yielded response rates that enabled surgery in approximately 70% of patients (^66^). Responses should be assessed early and every 1–3 months using cross-sectional imaging; surgery, performed by expert head and neck surgeons, should be planned as soon as resection is feasible (^4^) or earlier, based on a suspicion of progressive disease. The addition of immunotherapy to DT is discussed below (see the **Immunotherapy section**).

Evidence regarding the benefit of other BRAF/MEK inhibitors is limited. Their use may be considered when DT is unavailable (^97^).

### Other selective inhibitors

- **Recommendation 31:** If actionable fusions (*RET, NTRK, ALK*) are detected, treatment with selective inhibitors is recommended under tumor-agnostic indications, despite limited evidence and generally lower observed response rates.

Approximately half of ATCs are non-*BRAF*-V600E-mutated ATC tumors and are associated with mutations in the *RAS*, *NF1*, *PIK3CA*, and *PTEN* genes (**Table 3**) (^35^). Currently, these mutations are considered non-targetable. In contrast, targetable gene fusions involving *RET*, *NTRK*, *ALK*, and *BRAF* are rare, with individual frequencies below 5% (^35^). Whether these tumors can be effectively treated with selective inhibitors remains an open question. The small number of ATC patients with gene fusions has limited proper investigation, and current data are scarce.

The best-studied fusion-specific inhibitor is the NTRK inhibitor larotrectinib. Reported pooled data from three phase I/II larotrectinib clinical trials (NCT02576431, NCT02122913, and NCT02637687) included 27 patients with *NTRK* fusion-positive thyroid cancer, 7 of whom had ATC (^98^). In the ATC cohort, the ORR was 27% (95% confidence interval: 4–71), the median PFS was 2.2 months (95% confidence interval: 0.9–NE), and the 12- and 24-month PFS rates were both 17%; these findings were driven by a single patient with poorly differentiated thyroid carcinoma who achieved a durable response (^98^).

The MD Anderson group analyzed *NTRK* fusion-positive ATC and showed that responses to first-line NTRK inhibitors are often shorter than those observed in DTC. Newer NTRK/ROS1 inhibitors (e.g., repotrectinib and taletrectinib) are currently under investigation, and available data suggest that first-line NTRK inhibitor monotherapy in ATC typically yields non-durable responses (^99^). Regarding ALK inhibitors, at least two case reports describe significant tumor responses to crizotinib (^100^,^101^). In one case, a remarkable response was subsequently achieved with ceritinib and brigatinib after crizotinib failure (^101^).

The phase I/II LIBRETTO-001 trial of the RET inhibitor selpercatinib included a small number of patients with *RET* fusion-positive ATC, with at least one achieving a durable partial response (≥18 months) and another achieving stable disease. In the broader cohort of previously treated *RET* fusion-positive thyroid cancers (which included these ATC cases), the overall response rate was 85.4% and the median duration of response was 26.7 months; however, ATC-specific efficacy data were not reported separately (^102^,^103^).

Overall, the available data are too limited to draw firm conclusions. Based on reported experiences, ATC with targetable fusions is extremely rare, and responses to fusion inhibitors, although possible, are variable and generally short-lived. Neoadjuvant therapy with selective inhibitors may be considered for unresectable ATC tumors harboring actionable gene fusions (^104^). Further research exploring other specific inhibitors and combination therapies is necessary before these treatments can be recommended as first-line options.

### Cytotoxic chemotherapy and multikinase inhibitors

- **Recommendation 32:** For patients with unresectable or metastatic ATC without targetable mutations, or when molecular testing or targeted therapies are unavailable, enrollment in clinical trials is recommended. If clinical trials are unavailable, recommended options include lenvatinib/pembrolizumab, cytotoxic chemotherapy, or best supportive care.
- **Recommendation 33:** Lenvatinib-based systemic therapy (preferably lenvatinib plus pembrolizumab) is recommended for patients with metastatic or unresectable, non-*BRAF*-mutated ATC in centers with multidisciplinary expertise. This is particularly relevant when clinical trials or molecularly targeted options are not available, provided that contraindications to antiangiogenic agents are carefully excluded and close toxicity monitoring is ensured.

Given the limited efficacy of conventional therapies in patients with ATC without targetable mutations, participation in clinical trials is strongly encouraged (^13^). Unfortunately, access to clinical trials in Latin America remains highly limited, representing a major barrier to the implementation of innovative therapies for ATC in the region.

Therefore, in the absence of clinical trials, the combination of RT and cytotoxic chemotherapy is generally recommended for patients with good performance status who wish to pursue aggressive therapy (^13^,^105^). However, treatment responses are infrequent and, when achieved, are typically short-lived. Given the complexity of management decisions in ATC, a careful discussion weighing the potential benefits and drawbacks of aggressive treatment is necessary.

Various cytotoxic regimens, most commonly incorporating doxorubicin, cisplatin, or taxanes (paclitaxel or docetaxel), have been employed in ATC (^106^,^107^) (**Table 4**). Doxorubicin (administered weekly or every three weeks) remains the only cytotoxic agent specifically approved by the FDA for this indication. Pemetrexed plus carboplatin has shown promise in patients with refractory thyroid cancer, including one patient with ATC who was successfully treated following third-line therapy (^108^).

Tyrosine kinase inhibition has emerged as a potential option for non-*BRAF*-V600E mutated ATC, although its activity as monotherapy remains modest. Among multikinase/antiangiogenic agents, lenvatinib is the best characterized. Prospective and real-world series show objective responses in only 11–15% of patients, with disease control rates ranging from roughly one-third to two-thirds of cases and a median OS generally below 12 months. These limited benefits led to its regulatory approval in Japan; however, other guidelines maintain a more cautious stance due to associated toxicity and the frequent aerodigestive tract invasion observed in ATC, which typically contraindicate its use (^13^,^88^,^109^–^111^).

In clinical practice, lenvatinib is increasingly combined with immune checkpoint inhibition (most often pembrolizumab) to potentiate antitumor immunity and improve the depth and duration of response. This strategy has already been incorporated into the NCCN algorithms for metastatic, *BRAF*-negative disease when clinical trials or molecularly matched therapies are unavailable (^58^,^112^,^113^). Nevertheless, current evidence is derived from small, predominantly single-arm cohorts, and responses remain notably less robust than those obtained with *BRAF/MEK* inhibition in *BRAF*-mutated ATC. Antiangiogenic toxicity is a major limiting factor.

In settings where pembrolizumab is unavailable or unaffordable, lenvatinib monotherapy remains an option, with the understanding that responses are typically less durable. Other tyrosine kinase inhibitors, including sorafenib, imatinib, gefitinib, and pazopanib, have not demonstrated consistent or clinically meaningful activity as single agents and are therefore not routinely recommended (^114^–^117^).

For patients with an Eastern Cooperative Oncology Group performance status of 3–4, end-of-life care discussions are paramount and generally preferred over systemic therapy. For patients with an Eastern Cooperative Oncology Group performance status of 0–2, end-of-life discussions remain a reasonable option; however, systemic therapy (initiated after multidisciplinary review) may offer superior symptom relief and an improved quality of life.

### Immunotherapy

- **Recommendation 34:** The addition of pembrolizumab, when available, is recommended in *BRAF*-V600E-mutated ATC, since responses to DT are often robust but typically short-lived. This strategy may help delay or overcome resistance and improve the likelihood of durable response.
- **Recommendation 35:** In patients with unresectable or metastatic ATC who have high PD-L1 expression, high tumor mutation burden or microsatellite instability and lack other actionable mutations, checkpoint inhibitors may be considered, preferably in the context of a clinical trial.
- **Recommendation 36:** In patients with non-*BRAF* V600E-mutated ATC harboring other targetable alterations, immunotherapy may be considered in combination with other selective inhibitors, if available.

Immunotherapy, particularly immune checkpoint inhibitors targeting the PD-1/PD-L1 axis, has emerged as a promising therapeutic option for ATC. ATC tumors frequently express immune markers such as PD-L1, and immune infiltration is a hallmark of the disease, which justifies the immunotherapeutic approach. PD-L1 expression is observed in approximately 11–28% of cases, and mismatch repair deficiency occurs in over 10% in some series, supporting a rationale for immunotherapeutic approaches. Tumor PD-L1 expression, tumor mutational burden, and mismatch repair status may help identify likely responders (^118^).

For patients lacking actionable alterations, single-agent spartalizumab demonstrated an ORR of 19% and median OS of 5.9 months, with higher PD-L1 expression correlating with better responses (^118^). Pembrolizumab has shown benefit in high tumor mutational burden or high microsatellite instability tumors (ORR 29%) (^119^). Dual inhibition with nivolumab plus ipilimumab achieved an ORR of 30% in a small ATC cohort (^120^).

Combination strategies integrating immunotherapy with targeted agents have shown encouraging results. In a phase II trial, atezolizumab with genomically matched targeted therapy (*BRAF* V600E variant: vemurafenib plus cobimetinib; *RAS*- or *NF1/2*- mutant: cobimetinib; non-*BRAF/RAS/NF1/2* mutant: bevacizumab) achieved a median OS of 19 months, with the *BRAF* V600E cohort reaching 43 months (^121^). The combination of selective inhibitors and immunotherapy has been more *commonly studied* in *BRAF*-mutated ATC. Although data remain limited, in patients with *BRAF* V600E mutation disease, multidisciplinary tumor boards should evaluate the feasibility of integrating anti–PD-1 agents, especially in those with high tumor burden or suboptimal response to DT alone.

Resistance to BRAF inhibitors, remains a significant clinical challenge (^122^). To overcome this risk, the MD Anderson group proposes an induction therapy following a dual-step approach: DT is initiated to achieve swift cytoreduction and relieve present or imminent compressive symptoms. Within approximately four weeks, an immune checkpoint inhibitor targeting the PD-1/PD-L1 axis is added to counteract or delay development of therapeutic resistance (^3^). The addition of pembrolizumab to DT (DTP) improved outcomes in a retrospective study of 94 patients with ATC (^123^). DTP increased median OS from 9 to 17 months (*p* = 0.037) and PFS from 4 to 11 months (*p* = 0.049). The neoadjuvant cohort achieved the most favorable results, with a median OS of 63 months and a 50% pathological complete response rate, which was significantly associated with longer survival (*p* = 0.018). In a multicenter phase II trial (NCT04675710), neoadjuvant DTP enabled R0/R1 resection in 97% of patient with *BRAF* V600E-mutated ATC, compared to a historical rate of 5%. DTP demonstrated improved resectability, PFS (13.9 months), and OS, supporting its adoption as standard-of-care in *BRAF-*mutated ATC when feasible (^124^).

Regarding other selective inhibitors, the combination of larotrectinib with immunotherapy was attempted in one patient with *NTRK* fusion-positive ATC but resulted in significant hepatotoxicity (^99^). Moreover, the MD Anderson experience with combining immunotherapy and other fusion-targeted inhibitors, such as selpercatinib (*RET* fusion) and alectinib (*ALK* fusion), has also been associated with hepatotoxicity (^3^)

**Figure 1.**
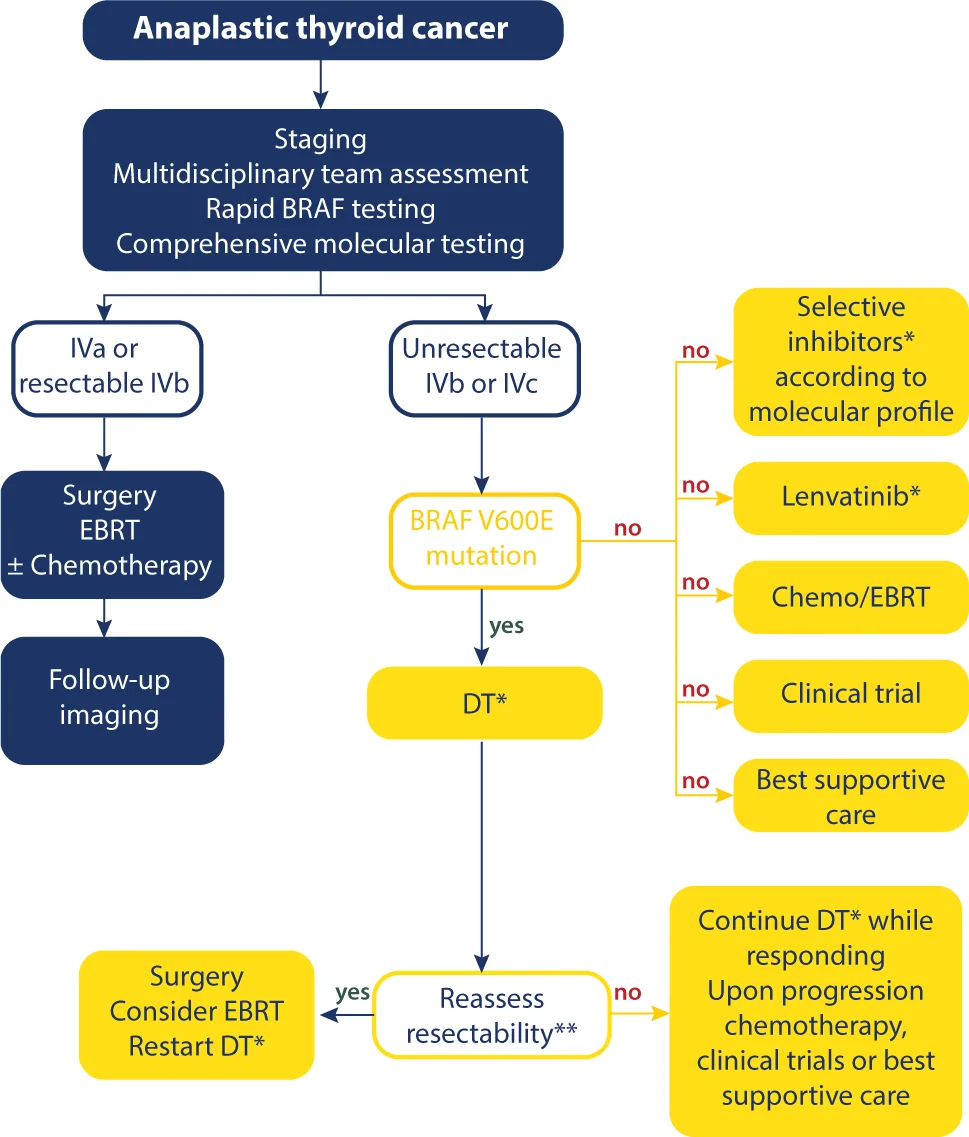
Management algorithm for anaplastic thyroid cancer.

An algorithm for the therapeutic management of ATC is shown in **Figure 1**.

*Immunotherapy; **Every 2-4 weeks.

DT: Dabrafenib/Trametinib; EBRT: external beam radiotherapy.

## OTHER ASPECTS

### Follow-up strategies

- **Recommendation 37:** ^18^F-FDG PET-TC (if available) or cross-sectional imaging should be periodically performed after initial therapy to assess disease status.

Due to its higher sensitivity, ^18^F-FDG PET/CT is the preferred modality for follow-up in patients with ATC. Nevertheless, in settings where PET/CT is not available, cross-sectional imaging (CT or MRI) of the neck, chest, abdomen, and pelvis—with measurement of target lesions to objectively assess disease status—may be used as an alternative. Additional imaging studies (e.g. brain MRI) should be obtained based on clinical indications. It is recommended that imaging follow-up be performed every 4–8 weeks after initial therapy (^12^), while PET/CT may be performed every 3–6 months (^58^). Patients receiving targeted therapies with neoadjuvant intent should undergo more frequent evaluations (every 4–6 weeks) to identify those with tumor responses who may become eligible for surgical resection (^12^). Symptomatic metastases (e.g., bone, brain) should be considered for local therapies, including EBRT, thermal ablation procedures, and/or treatment with bone antiresorptive agents such as intravenous bisphosphonates or denosumab (^13^,^58^).

## FINAL CONSIDERATIONS

Despite significant advances in the molecular characterization and treatment of ATC, translating these gains into real-world benefit remains particularly challenging in Latin America. In many countries across the region, access to comprehensive molecular testing, multidisciplinary care, and high-cost systemic therapies is inconsistent and frequently limited to a small number of referral centers. The availability of DTs in Latin America may be constrained by cost, regulatory hurdles, or supply issues, necessitating alternative approaches essential to ensure equitable care.

In a multicenter cohort study from Argentina, 67% of patients underwent molecular testing for *BRAF* V600E; however, fewer than 50% of mutation-positive cases received DT therapy (^125^). This gap between diagnostic capability and access to matched targeted treatment reflects a broader structural issue and underscores the urgent need to align guideline-based recommendations with local feasibility.

To address these disparities, region-specific strategies, including political and health policy initiatives are necessary. These strategies should involve advocating for the incorporation of DT into national formularies, implementing fast-track approval pathways for drugs with proven benefit in ATC, and promoting regional price negotiations, patient-access programs, and public–private partnerships. In parallel, strengthening referral networks, concentrating complex ATC care in experienced centers, and ensuring timely access to palliative radiotherapy, surgery, and optimal supportive care are critical steps. Lastly, establishing collaborative regional registries and pragmatic clinical studies is essential to generate real-world evidence regarding outcomes, toxicities, and patterns of care, thereby informing treatment algorithms and policy decisions. Implementing these strategies is essential to bridging the gap between what is clinically possible and what is realistically available to patients with ATC in the region.

## Data Availability

datasets related to this article will be available upon request to the corresponding author.
