# Erratum: Latin American Thyroid Society expert panel consensus on anaplastic thyroid cancer

**DOI:** 10.20945/2359-4292-2026-0116

**Published:** 2026-10-05

In the article: Califano I, Callegari FM, Cerutti JM, Chaves ALF, Castro G Junior, Ruiz-Tagle JMD, Falco A, Hoff AAO, Kowalski LP, Maia AL, Pereira MD, Sanabria A, Pitoia F. **Latin American Thyroid Society expert panel consensus on anaplastic thyroid cancer**. Arch Endocrinol Metab. 2026;70(5):e260100. doi: 10.20945/2359-4292-2026-0100.

Page 1, Authors

Where it reads:

Jose Miguel Dominguez Ruiz-Tagle^6^

https://orcid.org/0000-0002-6157-8055

Read:

Jose Miguel Dominguez^6^

https://orcid.org/0000-0002-6157-8055

